# Performance of Heart Failure Patients with Severely Reduced Ejection Fraction during Cardiopulmonary Exercise Testing on Treadmill and Cycle Ergometer; Similarities and Differences

**DOI:** 10.3390/ijerph182412958

**Published:** 2021-12-08

**Authors:** Reza Mazaheri, Mohammad Sadeghian, Mahshid Nazarieh, David Niederseer, Christian Schmied

**Affiliations:** 1 Department of Sports and Exercise Medicine, Division of Sports Cardiology, Tehran University of Medical Sciences, Tehran 1419733141, Iran; mazaheri_md@tums.ac.ir (R.M.); mahshid_md@yahoo.com (M.N.); 2 Department of Cardiology, Tehran Heart Center, Tehran University of Medical Sciences, Tehran 1411713138, Iran; msadeghian@sina.tums.ac.ir; 3 Department of Cardiology, University Heart Center, University Hospital Zurich, University of Zurich, 8091 Zurich, Switzerland; david.niederseer@usz.ch

**Keywords:** cardiopulmonary exercise testing, heart failure, peak VO_2_, treadmill, cycle ergometer, exercise test mode

## Abstract

Background: Peak oxygen consumption (VO_2_) measured by cardiopulmonary exercise testing (CPET) is a significant predictor of mortality and future transplantation in heart failure patients with severely reduced ejection fraction (HFrEF). The present study evaluated the differences in peak VO_2_ and other prognostic variables between treadmill and cycle CPETs in these patients. Methods: In this cross-over study design, thirty males with severe HFrEF underwent CPET on both a treadmill and a cycle ergometer within 2–5 days apart, and important CPET parameters between two exercise test modalities were compared. Results: Peak VO_2_ was 23.12% higher on the treadmill than on cycle (20.55 ± 3.3 vs. 16.69 ± 3.01, *p* < 0.001, respectively). Minute ventilation to carbon dioxide production (VE/VCO_2_) slope was not different between the two CPET modes (*p* = 0.32). There was a strong positive correlation between the VE/VCO_2_ slopes during treadmill and cycle testing (r = 0.79; *p* < 0.001). VE/VCO_2_ slope was not related to peak respiratory exchange ratio (RER) in either modality (treadmill, r = 0.13, *p* = 0.48; cycle, r = 0.25, *p* = 0.17). The RER level was significantly higher on the cycle ergometer (*p* < 0.001). Conclusion: Peak VO_2_ is higher on treadmill than on cycle ergometer in severe HFrEF patients. In addition, VE/VCO_2_ slope is not a modality dependent parameter and is not related to the patients’ effort during CPET.

## 1. Introduction

Peak oxygen consumption (peak VO_2_) is an important parameter to estimate prognosis and disease severity in heart failure (HF) patients with reduced ejection fraction. It is a determinative criterion in many treatment strategies decision making and a weighted factor in the majority of HF prognostic scores [1]. Cardiopulmonary exercise testing (CPET), which directly measures ventilatory gas exchange, is the gold standard to quantify peak VO_2_. It is a well-known method to provide precise and reproducible results, which evaluate the physiological response to progressive exercise [2].

The two most common exercise test modalities for CPET are treadmill and cycle ergometer. There is compelling evidence that peak VO_2_ values are higher on the treadmill but of various amounts (10–20%) according to the target population and the exercise test protocol [3,4]. This difference between the two procedures is sometimes crucial, such as in heart failure patients with severely reduced ejection fraction (HFrEF), where the value helps to stratify the patients whether heart transplantation is needed [5,6]. Previous studies in HFrEF patients are subject to several limitations, including diverse sample population from distinct centers with different exercise test modalities and protocols.

In addition to peak VO_2_, the percentage of predicted peak VO_2_ is another important parameter, especially in young patients (<50 years) and women [7]. There are some equations to calculate the reference predicted values of peak VO_2_, derived from age, sex, weight, height, and exercise mode. Notably, the weighting of the exercise mode is different among the equations, with a less than 2% variation in a recently proposed prediction equation [6] to 11% in Wasserman/Hansen equation [8]. To the best of our knowledge, there is no specific prediction equation for severe HFrEF patients in the literature.

Apart from peak VO_2_, ventilatory efficiency indicated by VE/VCO_2_ slope represents ventilation and perfusion matching within the pulmonary system and reflects disease severity and prognosis in HF patients. It is also a determining parameter for heart transplantation listing in the presence of sub-maximal testing [9]. This information implicates that the VE/VCO_2_ slope has a significant correlation with peak VO_2_ for risk stratification of the patients and should not be related to the patients’ effort, as it is still valuable in sub-maximal tests. As the respiratory exchange ratio (RER), which indicates exercise effort, is different between exercise modes in many prior studies [6,10], the assessment of its relation to other CPET parameters and its role in the interpretation of the test results is crucial.

The present study has been designed to evaluate the level of discrepancy between the two most popular exercise test modalities for peak VO_2_ measurement in severe HFrEF patients. The relationship between VE/VCO_2_ slope, peak VO_2_, and RER within and between each modality has been evaluated. The percentage of variation between peak VO_2_ on the treadmill and cycle attained from this study could be used for peak VO_2_ reference predictions in HF patients.

## 2. Materials and Methods

The present cross-over randomised clinical trial has been designed to compare the CPET parameters on the treadmill vs. cycle ergometer, the two most common modalities of exercise testing.

### 2.1. Study Population

Thirty male patients with stable heart failure (just mild signs and symptoms without recent change) and severely reduced ejection fraction (HFrEF) (EF ≤ 35%) were included in the study. They were referred from the university heart failure clinic, and all were on medical treatment for more than one year. The patients did not have an exacerbation of the disease and were not hospitalized for at least three months before enrollment. They were not considered for any intervention, such as revascularization or device therapy. Patients with chronic kidney disease and advanced pulmonary disease that could interfere with the results or any contraindication for exercise testing and limiting musculoskeletal problems were excluded.

The study complied with the Declaration of Helsinki and the research protocol was approved by the local ethics committee. The study process was explained to the patients, and an informed consent was signed.

### 2.2. Cardiopulmonary Exercise Testing

All participants underwent CPET on a treadmill and a cycle ergometer. They were randomly assigned to each mode of exercise for the first test, and the second test was done with the other modality within at least two and maximum five days apart (at the same time of the day, usually in the morning). An individualized ramp protocol was used for cycle ergometer tests with continuous increments of 5–20 Watt/min, depending on the patient’s abilities. Treadmill tests started with a speed of 2.7 km/h and progressed with 0.15 km/h and 0.5% grade every 30 s. The aim was to reach exhaustion and to achieve a test duration of more than 5 and less than 12 min [11].

Some information about medication use, food or caffeinated drinks, and physical activity restriction was given to the patients beforehand. Metabolic cart calibration was done before each test. Continuous 12-lead electrocardiogram and blood pressure measurement (every 2 min) were recorded during the tests. A commercial metabolic cart (Quarck CPET, CosMed, Rome, Italy) collected breath-by-breath gas analysis data, and the 10-s average outputs were considered for data analysis.

Peak VO_2_ (mL/kg/min) was expressed as the highest average value during the last 20 s of the exercise test, and the percentage of predicted peak VO_2_ was calculated based on the predicted values from the Wasserman’s equation [12]. Oxygen (O_2_) pulse defined as VO_2_ (mL/min) divided by HR was expressed as mL/beat and measured at rest and peak exercise. The VE/VCO_2_ slope was determined from the collected gas analysis data except for the initial seconds and the exaggerated ventilatory response during the last seconds of the tests. The maximum PETCO_2_ response was considered as its highest amount during the test, which is usually around the ventilatory threshold (VT).

The recovery phase of the tests was conducted in a sitting position, and the recovery heart rate (HRR) was obtained at the end of the first minute. The level of perceived exertion was inquired by a standard Borg scale (6–20 score) at the beginning of the recovery phase.

### 2.3. Statistical Analysis

A commercially available software (IBM SPSS Statistics for Windows, Version 25.0, Armonk, NY, USA: IBM Co.) was used for data analysis. Continuous quantitative variables are expressed as the mean ± standard deviation. Numerical data are expressed as numbers and percentages. The paired *t*-test was used to compare continuous data. Correlations between peak VO_2_ and the VE/VCO_2_ slope in each exercise modality and for each parameter among the two modes of exercise testing were evaluated by calculating the Pearson correlation coefficient. It was used to assess the correlation between VE/VCO_2_ slope and RER in each exercise mode as well. A *p*-value of ≤0.05 was considered as statistically significant.

## 3. Results

### 3.1. Baseline Characteristics

Thirty severe HF patients underwent CPET on both the treadmill and cycle ergometer. The baseline characteristics of the patients are shown in Table 1, and their comorbidities alongside their medications are demonstrated in Table 2. There were no changes in the patient’s baseline features between the test days. Two-thirds of the patients were diagnosed as idiopathic dilated cardiomyopathy, and the other one-third had ischemic heart disease. All patients were on β-blocker therapy.

### 3.2. Peak O_2_ Consumption and Associated Parameters

**Figure 1:**
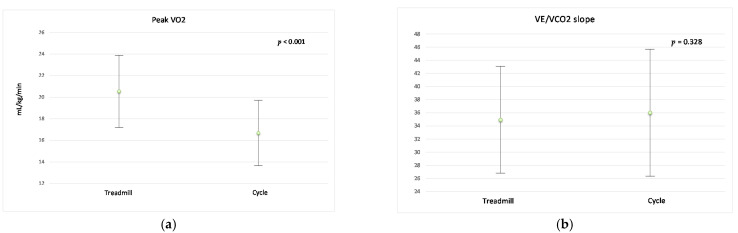
Differences of peak VO_2_ (**a**) and VE/VCO_2_ slope (**b**) between the exercise modalities.

Peak VO_2_ was 23.12% higher on the treadmill than on the cycle ergometer (*p* < 0.001). The percentage of predicted peak VO_2_ and oxygen uptake efficiency slope (OUES) were significantly higher on the treadmill as well. Peak VO_2_ values were well correlated between the two modes (r = 0.71; *p* < 0.001). Figure 1 shows the significant difference of peak VO_2_ (SD) between exercise testing modes. Despite equal baseline O_2_ pulse between the two CPET modes, the maximum O_2_ pulse was significantly higher on the treadmill as compared to cycle ergometer (*p* < 0.001).

### 3.3. Ventilatory Efficiency

**Figure 2:**
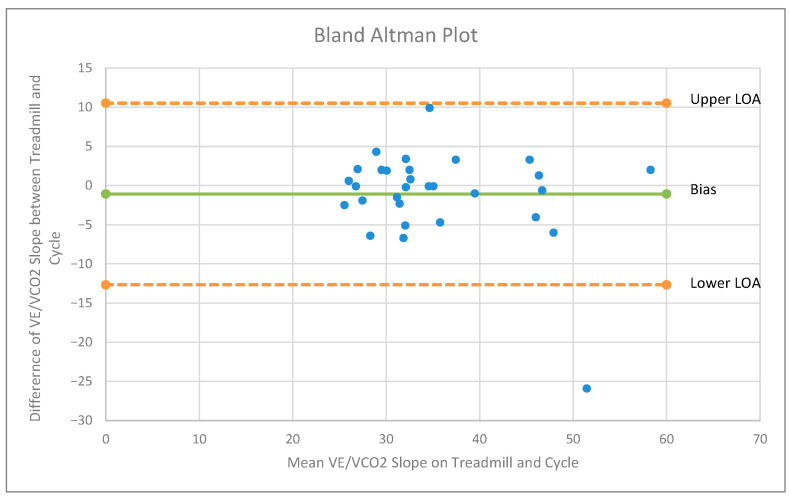
The level of agreement for VE/VCO_2_ slope between treadmill and cycle tests with 95% confidence interval.

VE/VCO_2_ slope and maximum PETCO_2_ response to exercise were not significantly different between the two modes of CPET (*p* = 0.32 and *p* = 0.47, respectively). There was a strong positive correlation between the VE/VCO_2_ slopes during treadmill and cycle testing (r = 0.79; *p* < 0.001). The level of agreement (LOA) between treadmill and cycle tests for VE/VCO_2_ slop is shown in Figure 2. There was a significant negative relationship between peak VO_2_ and VE/VCO_2_ slope on both exercise modes (treadmill, r = −0.52, *p* = 0.003; cycle, r = −0.44, *p* = 0.014). VE/VCO_2_ slope was not related to peak RER (treadmill, r = 0.13, *p* = 0.48; cycle, r = 0.25, *p* = 0.17).

### 3.4. Subject’s Effort

The RER level was significantly higher on the cycle ergometer (*p* < 0.001). Maximal and recovery heart rate showed almost the same response with each modality, without any significant differences. There were also no significant differences between the duration of an exercise test and the level of perceived exercise burden indicated by the Borg scale (6–20 score) on the treadmill and cycle. Table 3 shows the comparison of CPET parameters on the two exercise test modes.

## 4. Discussion

The most striking and impressive finding of the study is the fact that exercise testing of severe HFrEF patients on a treadmill leads to 23% higher values of peak VO_2_ than on cycle ergometer. This difference is more than previously reported in other populations. On the other hand, the ventilatory efficiency of the patients indicated by the VE/VCO_2_ slope and PETCO_2_ response to exercise did not show any significant difference between the two exercise test modalities. Interestingly, the VE/VCO_2_ slope was not related to the subjects’ effort identified by RER. The maximum oxygen pulse, reflecting stroke volume response to exercise, was higher on the treadmill, which is in line with the peak VO_2_ measurements.

### 4.1. Peak Oxygen Consumption

There is compelling evidence supporting the finding of higher peak VO_2_ values measured on treadmill tests than cycle ergometer; however, these differences have been shown only in a range between 5–10% [9] to 10–20% [13,14] and 5–20% [4]. These variations are most likely explained by inter-study and inter-individual variabilities. Compared to treadmill testing, untrained individuals usually terminate cycle tests at lower workloads due to quadriceps fatigue; thus, they tend to produce a lower peak VO_2_. Smaller and deconditioned muscle mass in a HF patient unexperienced to cycling might pronounce these differences compared with healthy individuals. 

In a randomised crossover study in HFrEF patients, Page et al. reported a significantly higher peak VO_2_, percentage of predicted peak VO_2_, and O_2_ pulse on the treadmill with a non-significant difference in VE/VCO_2_ at VT, maximum HR, respiratory exchange ratio, and perceived level of fatigue compared to cycle ergometer tests [15]. Except for the RER levels, the results of their study are in line with our findings. They found a higher peak VO_2_ of about 10% on the treadmill than on cycle ergometer. However, it seems that the significantly longer duration of the exercise tests on the treadmill (which was more than 13 min on average) forced the patients to stop the test due to their low endurance state, and higher values could have been achieved with shorter standard exercise test durations.

In a prospective study on patients with clinically mild HF, Maeder et al. demonstrated higher peak VO_2_ of about 10% on a treadmill as compared with a cycle ergometer [16]. VE/VCO_2_ slope values and HRR within the first minute of exercise were not different, but HRR within the second minute as well as RER were lower on the treadmill than on cycle. The authors did not find any significant inverse relationship between the two well-documented prognostic parameters in HF patients, peak VO_2_, and VE/VCO_2_ slope on cycle tests, so their findings are possibly limited due to low study power and a small number of participants.

Studies by Myers et al. [11] and Witte et al. [17] both illustrated a 16% higher peak VO_2_ on the treadmill compared to cycle ergometer. Variability of the patient’s characteristics in the Myers study (CAD and CAD with angina in HF patients) and low sample size in the Witte study (11 subjects) were limiting factors. The latter study showed a difference of more than 27% in peak VO_2_ (45.9 ± 13.2 vs. 36.0 ± 9.5 mL/kg/min) between the two exercise test modalities in the control group. Another study by Strzelczyk et al. found 18% higher peak VO_2_ values on a modified Naughton treadmill protocol compared to cycle testing in HFrEF patients referred to as potential cardiac transplant candidates [18].

The importance of correct assessment of peak VO_2_ and percentage of predicted peak VO_2_ as prognostic factors and for decision finding are well known [5,6]. In a study by O’Neill et al., peak VO_2_ was a predictor of mortality or future transplantation in HF patients. Each 1 mL/kg/min decrease in peak VO_2_ resulted in an adjusted hazard ratio of 1.25 (*p* < 0.0001) in their HFrEF patients receiving β-blockers [7]. Peak VO_2_ is a crucial parameter in HF survival scores, which are determining tools regarding referral to heart transplantation, particularly in HF patients with device therapy [19]. This evidence shows the importance of accurate calculation of the reference predicted peak VO_2_ values in each exercise test mode. For the calculation of predicted values on each modality, Wasserman’s equation considers an 11% difference [2], and the recently published FRIEND equation reflects just less than 2% variation between treadmill and cycle ergometer tests [9].

These dissimilarities in the measurement of peak VO_2_ and the differences in the prediction equations on different exercise modes imply an urgent need for a decisive approach, particularly in severe HFrEF patients. Determining a preferred method to measure peak VO_2_ and a valid equation to calculate the reference predicted values are essential. An accurate interpretation of the CPET results and the detection of a correct cut off value for peak VO_2_ could be performed afterward.

### 4.2. Ventilatory Efficiency

The VE/VCO_2_ slope and PETCO_2_ responses to exercise are indicative of disease severity as well as prognosis in HF patients [8,9]. There is evidence to advocate a more pronounced impairment of ventilatory efficiency during treadmill as compared to cycle exercise in patients with idiopathic pulmonary arterial hypertension [20]. Pulmonary gas exchange inefficiency and ventilation/perfusion mismatch during exercise may cause a higher ventilatory drive, with regard to carbon dioxide production. Witte et al. showed that the VE/VCO_2_ slope was significantly steeper on the treadmill than on cycle ergometer in chronic HF patients but not in the control group [21].

To compare the prognostic value of CPET parameters obtained from treadmill tests with cycle ergometer, Arena et al. investigated 207 HF patients from two independent centers and observed similar prognostic characteristics for both peak VO_2_ and VE/VCO_2_ slope between the two groups [18]. However, they did not find a significant difference in the VE/VCO_2_ slope between the two modalities of exercise testing. The study by Maeder et al. also showed a non-significant difference between the two modes of exercise [10].

In line with previous studies, we did not find any significant difference for VE/VCO_2_ slope and PETCO_2_ response between the two exercise test modes. These findings were not dependent on the subject’s effort in each mode as well. Therefore, our findings are in accordance with the evidence that underlines the significant prognostic value of VE/VCO_2_ slope in HFrEF patients irrespective of peak RER [11]. In our study, we found a significant inverse relationship between peak VO_2_ and VE/VCO_2_ slope, which confirms them as two independent prognostic markers.

### 4.3. Exercise Test Intensity Measures

The most valuable indicator of an individual’s effort in CPET is peak RER [9,22]. It is defined as the VCO_2_/VO_2_ ratio, and generally, a level of ≥1.00 is accepted to indicate sufficient effort [23]. In previous studies [10,11,12,17] except for the Witte et al. data [5], there was a significant difference in RER levels between treadmill and cycle tests. Our results are in agreement with these findings, demonstrating a significantly lower RER on the treadmill than on cycle ergometer at the same level of the hemodynamic response.

Heart rate response to exercise is affected by inherent inter-individual variability, and this variation is higher in patients with cardiac disease who take cardio-active medications [18]. Moreover, peak HR is used to calculate other CPET parameters (e.g., peak O_2_ pulse), so it is reasonable to evaluate its response on different exercise test modes. In this study, again in line with former studies [11,24], there was no significant difference in peak HR between treadmill and cycle tests. Similar results were found with regards to the first-minute recovery heart rate in our study.

In HFrEF patients with a peak RER < 1.0, peak O_2_ pulse is still related to future adverse events [17]. Due to higher peak VO_2_ and similar peak HR on treadmill tests, it is plausible to have a higher peak O_2_ pulse on a treadmill than on a cycle test. In accordance with Page et al. data [22], the results of our study confirmed a significantly higher peak O_2_ pulse values on the treadmill. In line with this inference, OUES, another parameter suggesting myocardial function [16], was significantly higher on the treadmill as well.

Maximal exercise testing is determined by the subjective symptoms of exhaustion rather than attaining a predefined percentage of maximal heart rate. The assessment of perceived exertion (RPE) using the Borg scale (6–20 scores) demonstrated the same levels on the treadmill and cycle tests. The results of the previous studies on RPE are in agreement with our findings [16,25].

### 4.4. Study Limitations and Strengths

The peak RER level, which represents exercise effort, was lower than 1.0 on our treadmill tests. As we also evaluated the patients’ effort during exercise by the Borg scale and peak HR, and there was no difference between the two exercise modalities, the lower RER levels might be gained due to the inherent physiological differences and muscle fiber recruitment during walking and cycling [18]. In many previous studies, the level of RER was lower on the treadmill than on the cycle ergometer. Since the average RER was 1.01 ± 0.11 on cycle tests, it is reasonable to accept an RER of lower than 1.0 on treadmill tests.

Moreover, if the patients were obliged to gain a higher RER on the treadmill, the peak VO_2_ difference would be even higher than the presented findings, which implies the importance of exercise test mode on the interpretation of CPET results, particularly in severe HFrEF patients. In a study on moderate to severe HF patients, Beckers et al. attained suitable RER levels (>1.25) on both cycle ergometer and treadmill. They concluded that the mode of test significantly affects peak VO_2_ values, and it should be taken into consideration in exercise prescription [26].

The study population were heart failure patients with severely reduced EF and were not candidates for revascularization, so the number of patients with non-ischemic cardiomyopathy was more than ischemic HF patients. Indeed, the majority of patients were evaluated whether heart transplantation would be indicated in the future.

The cross-over design of the study, with the same patients for both exercise testing modes, is a definite strength of the present work. The tests were performed within a maximal time frame of five days, and many inter-individual variations and different metabolic carts with diverse calibration methods in multi-center studies could be excluded by our study design.

## 5. Conclusions

The main finding of the present study was a 23% higher peak VO_2_ on the treadmill compared to cycle CPETs in severe HFrEF patients. The VE/VCO_2_ slope did not significantly differ between the two exercise testing modalities, and it is not related to the patients’ effort during the test. RER was lower on the treadmill despite similar subjects’ effort and peak HR with both exercise modes.

## Figures and Tables

**Table 1:** Baseline characteristics of the patients.

	Mean (SD)	Range
Age (years)	45 ± 12	19–65
Height (centimetres)	172 ± 9	158–193
Weight (kilograms)	73 ± 19	49–117
BMI (kg/m^2^)	25.3 ± 5.1	18.3–39.4
LVEF (%)	18.7 ± 6.7	10–35

**Table 2:** Comorbidities and medication of the patients.

		No (%)
Clinical conditions and Comorbidities	Dilated cardiomyopathy	20 (70%)
	Ischemic heart disease	10 (30%)
	Current Smoking	8 (28%)
	Hypertension	4 (13%)
	Hyperlipidemia	2 (7%)
	Diabetes	1 (3%)
Medication	β-blockers	30 (100%)
	Diuretics	29 (97%)
	ACEIs/ARBs	28 (93%)
	Statins	17 (57%)
	Nitroglycerine	7 (23%)
	Phosphodiesterase inhibitors *	3 (10%)

* PDE-5 inhibitor (sildenafil).

**Table 3:** Cardiopulmonary exercise testing parameters.

CPET Variable	Treadmill	Cycle	*p*-Value *
Peak VO_2_, cc/kg/min	20.55 ± 3.34	16.69 ± 3.01	<0.001
Percent Predicted Peak VO_2_, %	58 ± 14	52 ± 15	0.001
Oxygen Uptake Efficiency Slope (OUES)	2525 ± 753	1873 ± 626	<0.001
O_2_ Pulse Rest, mL/beat	8.13 ± 2.76	7.64 ± 2.23	0.153
O_2_ Pulse Max mL/beat	12.85 ± 3.6	10.8 ± 2.82	<0.001
VE/VCO_2_ Slope	34.94 ± 8.13	36.02 ± 9.68	0.328
PET CO_2_ Max, mmHg	34.26 ± 5.59	34.62 ± 4.7	0.478
Peak RER	0.94 ± 0.09	1.01 ± 0.11	<0.001
Peak HR, beat/min	126 ± 14	121 ± 15	0.060
Recovery HR 1 min, beat/min	15 ± 10	17 ± 8	0.105
Exercise test duration, second	293 ± 94	291 ± 72	0.891
Borg Scale (6–20)	13.8 ± 1.8	13.5 ± 1.4	0.508

* Paired *t*-test.

## Data Availability

The data are not publicly available.
